# Hospital and Institutional News

**Published:** 1915-09-11

**Authors:** 


### HOSPITAL AND INSTITUTIONAL NEWS.

### A LEAD FROM LIVERPOOL.

At the instigation of Lord Derby, and under his chairmanship, a joint committee has been formed of the Liverpool branches of the Red Cross Society, the St. John Ambulance Association, and the West Lancashire Territorial Nursing Service Committee. There is no intention to interfere with the independence of each Association, and the new committee will not seek to raise money, its object being to compare notes, make suggestions and recommendations, secure combined action, and prevent overlapping. Other local voluntary organisations, not constituted under Government authority, such as the Liverpool Civic Service League and the Women's War Service Bureau, will be invited to join forces with the new committee.

### A HAPPY INSPIRATION.

There was a very pleasant ceremony during Sir Robert Borden's recent visit to France, this being in connection with the offer by the Canadian Prime Minister to the French Government, on behalf of Canada, of a hospital for the treatment of French wounded. The offer was accepted with gratitude, and it is hoped that the hospital will soon be opened near Paris. There will be accommodation for about five hundred patients, and the staff will consist of French Canadians, under the direction of Colonel Mignault, of Montreal, who has been recently engaged in hospital work in this country. The hospital will be carried on in the open air, but it will be working all the year round. One more evidence of the supreme virility and good will of Canada and Canadians.

### HONORARY (WAR) DEGREES.

The University of London has decided to confer an honorary degree of B.A. on internal students who have spent less than nine months in approved war service or been invalided in such service. The conditions are that the students must have spent not less than two years in study in the university, have passed the intermediate and second-year College Examination, and been certified by their teachers as likely under ordinary circumstances to have passed the final test. The *Journal of Education*, which publishes the above information, remarks that it is not clear whether students who passed the intermediate while still at school will be eligible. The degrees thus conferred will be clearly distinguished from those gained by examination, they will be recorded in special lists, and will presumably have a special name. Medical students are excluded, because medical degrees carry the right to practise.

### HOW IS IT DONE?

The papers mention that the York County Hospital has been "labouring for years under a debt of £3,900." If this be so, it demonstrates either great incapacity in the management or unique meanness on the part of the citizens of York. It is announced, further, that the York County Hospital has been called upon by the military authorities to undertake further duties and additional expenditure. In consequence the committee have placed 120 beds for wounded soldiers at the disposal of the War Office. The number of beds in this hospital is returned as 155, of which 116, by the recent returns, were constantly occupied by the civilian population. The committee, however, announce that the placing of 120 beds at the disposal of the War Office will not interfere "with the claims of the civilian population." We should welcome an explanation of how it has been possible to apply the principle of Pandora's Box to the bed accommodation at the York County Hospital.

### BOB SAWYER AT HIS BEST.

We can imagine no finer epitaph to a fallen hero than the letter written to the mother of Lieut. Edgecumbe Procter, of the Norfolk Regiment, who was recently killed in action. Lieut. Procter, who was the son of a medical man, wished to become a medical missionary, and with that object in view he entered Middlesex Hospital as a student, leaving it shortly after war broke out, when he obtained his commission. After his death his mother received from a sergeant in his company a letter in which occurred the following beautiful passages: "You cannot imagine how much we miss him. We are from all ranks of life, and each and every one of us looked to him as our leader and our hope and worshipped the ground he trod on. When I found he had been shot through the left eye and right through the brain, I believe I went mad. I carried him down to the trench and he gasped his last. My God! I and every man in the whole company would gladly have sacrificed all our lives if we could only have saved him." A hospital should, indeed, be proud of its students who can command such respect as this.

### POOR-LAW OFFICERS.

The Poor-Law Institutional Assistant Officers' Association has drawn from the President of the Local Government Board an important pronouncement on the activities of the Departmental Committee now inquiring into the appointment, duties, and remuneration of Poor-Law officers. The secretary of the Association wrote to Mr. Long pointing out that the large number of Poor-Law officers serving with His Majesty's Forces would have no opportunity of putting their views before the Committee through their Associations, and that of those remaining in the service many were so busily engaged in war work of various kinds that they had no time to prepare evidence for submission. In his reply Mr. Long stated that "while the Departmental Committee have not found it necessary to suspend their inquiry altogether, he understands that they do not propose to issue any further report affecting the position of institutional or other Poor-Law officers before the close of the war."

### WOMEN IN MILITARY HOSPITALS.

The valuable work that is being done by medical women in connection with the treatment of wounded soldiers was referred to at some length in The Hospital last week. It was shown how at least one large military hospital is conducted by women, and the success achieved by Dr. Flora Murray and her staff suggests that if women can be so valuable in the higher grades of the work of military institutions, they can be at least as useful in other ranks. It is therefore not surprising that a scheme is being inaugurated for women of the Voluntary Aid Detachment of the British Red Cross Society to take the places of men in military hospitals. Under this scheme they will be employed in the room of male orderlies, acting as dispensers, clerks, storekeepers, and cooks, and thousands of men may thus be set free for active service. Women thus engaged in military hospitals need not have the first-aid or nursing certificates, but must belong to a Voluntary Aid Detachment. There is, of course, nothing novel in the idea as applied to civil life, and there appears to be no reason why its application in the case of military hospitals should not be just as successful.

### SANATORIUM BENEFIT IN NORTHAMPTON.

The Northampton Insurance Committee has recently come to what is apparently an advantageous understanding with the Northampton Town Council in regard to the administration of the anti-tuberculosis scheme for that town. An agreement with the council has been made which provides that, in consideration of an annual payment at the rate of 7d. per head of the insured population, the Town Council shall offer the services of the tuberculosis officers, provide treatment at the tuberculosis dispensary, in sixteen beds in sanatoria and in eleven hospital beds, and furnish outdoor shelters, drugs, medicinal foods and other accessories, exclusive of nourishment, as may be deemed necessary by the tuberculosis officers. Whatever may be said of the action of the Public Health Committee during the past seventeen years, it now seems to be moving with considerable earnestness towards grappling systematically with the tuberculosis problem in Northampton. In this town there is a movement on foot to provide a separate sanatorium for consumptive workers in the boot trade; statistics show that the incidence of the disease is heavy among the employees in the staple trade of the town.

### INFANTS AND DRINK.

Everyone has heard of the lady " with a past," but in East Ham it appears that even infants may admit a similar unhappy record. At all events, the medical officer of the district announces the presence in the hospital of a child of one year and ten months who regularly calls for his beer, mistaking, presumably, the nurse for a Hebe of another quality. The suggestion, of course, is that " in days that were earlier " the unfortunate youngster was lured on to beer, if not to skittles, by maternal example and exhortation. There would seem to be ground for this in the experience that, while children may not be taken inside a public-house, some of the East Ham mothers circumvent the law by gathering at the familiar corner and drinking not within, but outside, the establishment. To these entertainments they bring their families, or at least the infantile portion of them, so that babies, beer, and bronchitis follow one another in due sequence. Among themselves, so it is alleged, the women and the publicans so arrange matters that no discovery takes place, and no holder of a licence has been prosecuted during the year. Thus what seems *prima facie* a testimony to character is really a proof of mere dexterity in deceiving the officers of the law. The picture is not a pleasant one in a city which claims to be the heart of the Empire.

### THE BRITISH ASSOCIATION.

As Mr. Balfour wrote in his graceful letter to Sir Henry Roscoe, the British Association " meets this year under circumstances which deprive the gathering of much of its social charm." But despite the absence from the programme of the festive functions, which are usually such pleasing adjuncts to the meeting, there is every promise that its scientific labours will be not less fruitful than in years gone by. The opening session was held on the evening of September 7 in the Free Trade Hall, Manchester, and at the outset Professor Arthur Schuster, the president, read a message to the King, expressing the devoted loyalty to His Majesty of the members of the Association, and assuring His Majesty that the Association as a whole and every individual member thereof are whole-heartedly anxious to devote all their energies to assisting the Government in the task of bringing the war to a victorious conclusion. Many of the members of the Association, probably the majority, have placed their services at the disposal of the Government for the purposes of the war, and, although the papers communicated during the sessions of the eighty-fifth annual meeting are not likely to relate to the work the individual members are doing, it is well that this fact should be placed on record.

### PROFESSOR SCHUSTER'S ADDRESS.

In his address as President of the British Association Professor Arthur Schuster dealt with a subject which is better understood by the public generally than the subjects of presidential addresses usually are. The title he gave it was " The Common Aims of Science and Humanity," and at the outset he showed how the Association had been able to exercise a powerful influence on the progress of science. But, he went on to say, the time had now arrived to consider whether it might be possible to secure not only greater continuity in the work of the Association, but better co-ordination with that of other scientific organisations. In this connection it is important to note that it is Professor Schuster's intention to endeavour to give effect to his views through the appointment by each section of the Association of a committee to advise the Council in science matters connected with the prosecution of the war. Indisputably the present crisis affords an opportunity for the Association to adapt itself to the needs of the times. Incidentally the President alluded to the need for the closer application of scientific methods to business, and showed how it was now being recognised that commerce might greatly benefit from intercourse with pure science. But Professor Schuster's address was too comprehensive to be summarised in a brief space, and we advise our readers to take an early opportunity of reading the full text of this admirable discourse.

### HUMOURS OF TYPHUS.

Of the horrors of typhus in Serbia we have heard much, though doubtless far from the whole story. A writer in one of our contemporaries relates some of the "humours" of the situation, though, truth to tell, they hardly reach a very high level of entertainment. One of the most telling is an experience of a journey in a specially disinfected train, which, to judge from the record, had been practised on by someone more vigorous than discreet. The carriages were filled with some form of vapour, the odour of which was so acrid that eyes and noses wept in concert and the passengers struggled for the privilege of travelling with their heads out of the windows. Some amusement, too, may be discovered in the two typhus patients who competed with one another in the matter of temperature, and who, one one occasion, showed an equal record; here there being no possibility of victor and vanquished the argument was carried to other planes, and finally led to a hand-to-hand struggle, with a knock-out blow for the weaker rival. During the epidemic, we are told, the nurses were compelled to wear Turkish trousers, and some of them rebelled, but all in vain.

### THE "MILNE" TREATMENT AGAIN.

The efforts of Dr. Robert Milne to convince the medical profession at large, and the superintendents of fever hospitals in particular, that his now well-known method of treating scarlet-fever patients is safe and successful do not seem as yet to be crowned with a notable measure of success. He has recently circulated a fresh pamphlet relating to the method he uses in Dr. Barnardo's village homes. In this appears several photographs showing how cases of scarlet fever are allowed to mix freely with other children, among whom were surgical cases. The two provisos laid down by Dr. Milne are, first, that the treatment must be commenced early; secondly, the treatment must be carefully carried out. In connection with the first proviso, an early diagnosis is essential, and Dr. Milne describes certain appearances in the fauces which he states may be seen as long as twenty-four hours or even forty-eight hours before the rash is visible on the skin. Dr. Milne is in an advantageous position in this respect in dealing with numbers of children directly under his watchful eye; the general practitioner and the physicians in isolation hospitals rarely would have the opportunity of investigating this point. We discussed the whole question in The Hospital a year or two ago in connection with a Scotch isolation hospital, whose superintendent came into conflict with his Board on matters arising out of it.

### THE TYRANNY OF THE REGULATION.

The well-being of all organised communities depends upon the enforcement of definite regulations. But full success in administration is reached only by the man who knows when to allow departure from the rules. The hardships which may fellow the hard-and-fast application of a reasonable rule are illustrated by a discussion at the last meeting of the Holbeach Board of Guardians. A complaint was made that the mother of a patient, whom the doctor had said could not live through the night, was ordered to leave at ten o'clock because there was a rule that no visitor should remain later. The Guardians decided that it should be left to the discretion of the master to allow one relative to stay through the night with a dying patient. A medical man would have had no hesitation in using his discretion in a case like this whatever the rule might be. In that attitude lies the difference between the man accustomed to deal with the realities of life for which it is difficult to formulate rules and the man who deals in words and figures. The order of the Guardians permitting a variation of their regulation is obviously right. We think, however, the decision should not be left to the discretion of the master. The matter should be entirely in the hands of the medical officer, and only when his decision cannot be obtained should it be referred to the master.

### MEDICAL STUDENTS IN WAR-TIME.

An interesting article appeared in the Educational Supplement of the *Times* on the large decrease in the number of medical students, and the following table, which shows approximately the number of students in certain medical schools during the first year of war compared with the normal figures, is published:—

| — | Normal Numbers | 1914–1915 |
|---|---|---|
| St. George's ... ... ... ... | 80 | 20 |
| St. Bartholomew's ... ... ... | 450 | 285 |
| King's College Hospital ... ... | 70 | 45 |
| King's College ... ... ... ... | 232 | 107 |
| Cambridge ... ... ... ... | 164 | 111 |
| Manchester ... ... ... ... | 300 | 250 |
| Leeds ... ... ... ... ... | 150 | 100 |
| Liverpool ... ... ... ... ... | 166 | 123 |
| Aberdeen (men)... ... ... ... | 300 | 250 |
| Glasgow (men) ... ... ... ... | 650 | 600 |
| Total ... ... ... ... | 2,562 | 1,891 |

A further serious fall in numbers is anticipated by the majority of medical schools for the term which opens next month, and under these circumstances it is all the more difficult to understand the attitude of the War Office, alluded to in The Hospital last week, in countenancing the taking of combatant commissions by junior medical students.

### A SANATORIUM AND THE LIGHTING REGULATIONS.

The secretary of the East Anglian Sanatorium at Nayland was recently summoned for failing to comply with the orders for subduing the lighting at this institution, which it was alleged was too powerful, and he was fined two pounds. The report shows that there was a good deal to be said on the secretary's side, seeing that a special constable, who stated that his business sometimes took him to the sanatorium four nights a week, deposed that he had never seen a bright light at the sanatorium, and that since the order had come into force there had been scarcely any light at all. It appears that according to the police evidence a number of complaints had been received from persons travelling in the direction of Colchester by train, that the lights at the institution were clearly visible. It is only fair to the police to say that they sent to the secretary a warning in May, which was duly acknowledged. This case illustrates the supreme importance attaching to the enforcement everywhere of the most scrupulous care on the part of all responsible for the management of institutions, public and private buildings, and houses, to see that all the lighting regulations are strictly complied with, despite the difficulties that may necessarily arise when lights have to be kept on during the greater part of the night.

### SCHOOL INSPECTION AT IPSWICH.

The annual report on the medical inspection of children in attendance at elementary schools in Ipswich recites experiences which are now familiar, and which closely agree with records from other sources. The report, however, is a particularly clear and businesslike document, and its authors—Dr. Pringle and Dr. J. Green—are to be congratulated on its production. A word of recognition may be offered to the proposal to summarise the report in a form suitable for distribution among the parents and other citizens. All these official documents have characters which repel rather than invite the non-technical reader. No doubt many of these characters are necessary for statistical and formal purposes, but none the less they alarm the layman. Yet it is certain that the full health value of medical school inspection, and, indeed, of many other matters connected with the public health, will not be attained until they are supported by public opinion. This support can only be gained by the diffusion of teaching, which, so far as the public is concerned, is largely buried under masses of figures and statistical tables. What is wanted is the removal of these hindrances and the presentation of the actual position in a form readily appreciated by the mass of the people. The Hospital has urged this step on many occasions, and we shall be delighted to see Ipswich setting a good example. To think that health measures, or, indeed, other disciplinary proceedings, can be imposed on a wide scale as a mere official act, and apart from the force of public opinion, is a profound political mistake; it argues blindness to what is one of the most striking features of the national genius.

### INSANITY AND THE WAR.

Among the most pathetic consequences of the war have been cases of mental disturbance produced in officers and men by the rigours of the trenches and by the horrors of the stricken field. Some of these cases belong to the neurasthenic or hysterical order, and special arrangements have been made for the treatment of the victims and for their protection from the stigma of certification and confinement in an asylum. But there have been definite cases of insanity occurring at the Front, and these, of course, have had to be met by appropriate measures. As a contrast to this picture it is pleasing to know that the influences of patriotic duty and of a call to service have had a stimulating rather than a depressing action on the general mental tone of the nation. Thus Dr. William Graham, resident medical officer of Belfast Asylum, comments on the small number of admissions to the asylum during the year, and ascribes the decrease to the bracing influences of the war. The record of heroic actions and the sense of obligation to a great cause tend to check mental brooding and sustained selfishness, and hence they neutralise influences which are depressing to the mind and nervous system. Yet, again, it must be remembered that it is not great tragedies which break down men and women so much as continued petty worries, sustained monotony, and a sense of hopelessness. War, indeed, has its horrors and miseries and wickedness, but it has a stimulating influence on the national life when it is sanctioned by a great cause and is pursued with determination and high resolve.

### TUBERCULOUS SAILORS AND SOLDIERS IN MIDDLESEX.

The Middlesex County Council whilst, as we remarked last week, experiencing difficulties owing to the absence of some of their tuberculosis medical officers, are fortunately situated with regard to certain of the needs of the tuberculosis campaign; and especially in facilities for dealing with sailors and soldiers invalided in consequence of tuberculosis disease. The Council, when initiating the county scheme, purchased the " old " Hounslow Hospital premises, which had been vacated upon the completion and occupation of a new building. These premises, which were admirably adapted for renovation and enlargement, were arranged as the central dispensary for the fifth area of the county; provision was also made for observation wards and for the necessary staff. Doubts have been expressed as to the economy and utility, in normal circumstances, of observation wards attached to a dispensary; when, however, there arose the urgent need for accommodation for sailors and soldiers who, being found to be tuberculous, could not remain in the naval and military hospitals, such wards as those at Hounslow assumed a new and special usefulness. The County Council were able at once to arrange for admittance to one of them of all such patients as belonged to the county. This unforeseen application of part of the accommodation of the " observation " wards at Hounslow is proving of the greatest value, and a very useful work is being done with a minimum of expense and delay.

### MEDICAL VICTIMS IN THE "ROYAL EDWARD."

Two Manchester hospital doctors are among the officers officially reported to be "missing, believed drowned," as a result of the loss of the transport, *Royal Edward*—Captain Charles Bertram Marshall of the 3rd East Lancashire Field Ambulance (T.F.) Royal Army Medical Corps, and Lieutenant Thomas Hayhurst, of the 1st East Lancashire Field Ambulance. Dr. Marshall, who was twenty-seven years of age, was the younger son of Mr. William Marshall, of Cheadle Hulme, and was educated at Manchester Grammar School. He took his medical course at Manchester University, obtaining his M.B., Ch.B. in 1909, D.P.H. in 1911, and M.D. in 1913. He afterwards held appointments as demonstrator in anatomy at the University, and house physician or surgeon at the Children's Hospital, Pendlebury, the Manchester Royal Infirmary, the Manchester Consumption Hospital, the Ancoats Hospital, and the Royal Infirmary, Bradford. He obtained a commission as an Army surgeon while at Bradford Infirmary, and, after training ambulance men at various camps in this country, was in June appointed to act as commanding officer of the 2/3rd East Lancashire Field Ambulance and detailed for work at the Dardanelles. Dr. Hayhurst was twenty-six years old, and the son of Mr. J. A. Hayhurst, of Mayfield, Fulwood, Preston. Educated at King William's College, he took his M.B. at Edinburgh University, afterwards serving at Victoria Hospital, Burnley, and as senior house surgeon at the Manchester Children's Hospital, Pendlebury. He was a well-known Rugby footballer.

### THE SUPINENESS OF CAMBERWELL.

Dr. H. W. Bruce, medical superintendent of the Southwark Infirmary, has again called the attention of his Board of Guardians to the alleged nuisance at the infirmary caused by the close proximity of a dust siding used by the Camberwell Borough Council. He points out in a report that, although there appears to be some diminution in the actual amount of refuse handled on the railway siding, the methods adopted remain the same, and the siding is littered with refuse and manure, affording unlimited breeding places for flies. Dr. Bruce states that the nuisance from smell and flies is again becoming manifest, with accompanying disease, for already ten cases of enteritis have occurred amongst the children. Dr. Bruce has again protested against this insanitary work being carried out in a manner dangerous to the health of the patients and in the immediate neighbourhood of the infirmary. Three years ago the Southwark Board of Guardians appealed to the Local Government Board to enforce a remedy, and finally they were referred by that Board to the London County Council, which after due delay and inquiry stated they would be prepared to take action against the Camberwell Borough Council if a specific case could be made out. Now that the Southwark Board have the necessary evidence which can be examined by the medical officers of the London County Council, the Guardians should forthwith direct the attention of that Council to the grave dangers caused by this scandalous nuisance and use all their forces to secure its removal once and for ever.

### THE NURSING DIFFICULTIES IN INFIRMARIES.

The great difficulties experienced by Boards of Guardians in obtaining not only adequate medical staffs but also nursing staffs is borne out by a report submitted to the Southwark Board of Guardians by their Infirmary Committee. Out of twelve head nurses only three remain, and of thirteen staff nurses only two, whilst the places of two night superintendents are being filled by temporary officers. There are at present only five trained nurses available for the sick inmates, and Dr. H. W. Bruce, the medical superintendent, has reported that it is impossible for him and the matron to accept the responsibility for the care of over 600 patients. It is further a fact that the probationers at Southwark Infirmary cannot receive the training to which they are entitled, for this training was the sole justification of the low salaries they are paid. The winter may find the infirmary depleted of trained nurses, for the salaries offered at the present time are totally inadequate to attract new candidates, or even to retain the present staff. An application to the Local Government Board to put the head nurses on the same scale of remuneration as those engaged in the military hospitals has been acceded to, so that the head nurses will be paid £40 per annum for the period of the war. A deputation of the Guardians has been appointed to wait on the Local Government Board to discuss what steps shall be taken to meet the very serious outlook. The Southwark Board of Guardians unfortunately are not alone in having to face similar difficulties, and the result of their interview with the Local Government Board is awaited with widespread interest throughout the service.

### NO ROOM FOR THE MENTALLY DEFICIENT AT WANDSWORTH.

The difficulty of the Asylums and Mental Deficiency Committee of the London County Council to obtain suitable places to accommodate patients in South London has been referred to in the columns of The Hospital. The committee, with a view to endeavouring again to overcome the difficulties, have made another appeal to the Wandsworth Board of Guardians, asking for their consent to use any premises they may have available suitable for patients mentally deficient. When this letter came to the notice of the Guardians at a recent meeting scant consideration was given to it, the whole subject being dismissed by the acting chairman, Mr. Percy Rees, in the short sentence that they had no vacancies, as the Guardians had to board out some of those under their care, and so they had no places to let. The Wandsworth Board of Guardians have done noble service in assisting the War Office, and have been put to considerable trouble and worry in order to meet the demands made upon them. It is disappointing that its members and officials have not exerted themselves to help another authority in a period like the present. It must be discouraging to the committee to be met with rebuff after rebuff in their efforts to do all they can for the poor unfortunate people committed to their care, and it would appear that the only way out of the difficulty, so far as South London is concerned, will be for the County Council to take premises, to equip them, and secure a sufficient staff to look after the patients until they can be removed to suitable institutions. This would be a somewhat costly scheme, especially in these days when public authorities are called upon to exercise the utmost economy, but the expenditure has been made urgent by the action taken by the Wandsworth Board of Guardians and the neighbouring Board of Guardians at Lambeth.

### FORTUNES IN PROPRIETARY ARTICLES.

In The Hospital of August 7 (p. 388) we had the pleasure of recording munificent bequests to charitable institutions by the late Mr. J. C. Eno, proprietor of "Eno's Fruit Salt." It is with pleasure we now record another series of interesting and useful bequests, under the will of the late Mr. Edwin Carr, a Leicester manufacturing chemist, and proprietor of Carr's fever powders. To the Leicester Royal Infirmary he bequeaths £5,000, to the Children's Hospital £2,000, to Dr. Barnardo's Homes £500, to the Leicester Association for the Welfare of the Blind £500, to the Wycliffe Society for the Blind (Leicester) £200, to the National Society for the Prevention of Cruelty to Children (local branch) £200, to the Leicester Guild of the Crippled £200, to the Leicester District Nurses' Association £200, to the Leicester Ragged School Mission £200, Leicester Mission to the Deaf and Dumb £200, and to the Leicester Charity Organisation Society, the "Fairè" Hospital, the Leicester Maternity Hospital, and the Boys' Home, Avenue Road, Leicester, £100 each. All the legacies are free of duty, and the Leicester Royal Infirmary participates to the extent of one-half in the distribution of the residuary estate. It is all to the credit of the voluntary hospitals and charitable institutions of Leicester that their work and administration should appeal so forcibly to men of keen business acumen, who have made fortunes from a side issue of the drug habit, that they should leave behind munificent bequests for the provision of medical and surgical treatment. In the case of Mr. Carr, his bequests are coupled with the wish that they should be capitalised, and in order to benefit still further the residuary legatees, his executors are empowered to postpone the sale of his securities and the payment of the legacies for a period of two years, and the residuary estate for a period of three years from his death or until after the war, directions which show that the financial stability of the Infirmary and Children's Hospital in the years to come was viewed with importance and interest.

### EUCALYPTUS ONCE MORE.

It is a grateful name is eucalyptus, and it reaches all our ears from time to time. Its most recent virtue has come by telegraph. The announcement, which is contained in a despatch from Melbourne, is to the effect that Dr. Richard Bull, director of the bacteriological museum in the University of that city, claims to have discovered in eucalyptus a specific remedy for cerebro-spinal meningitis. Doubtless this is true, considering that the germ is one which is easily destroyed, and that eucalyptus undoubtedly possesses definite anti-bacterial qualities. The problem in practice is not to destroy the germ, but to destroy it when present in the human body—that is, to kill the parasite without damaging the host. The recent experience of the disease in this country has not fully settled the question of treatment. There is, however, general agreement that repeated lumbar puncture is beneficial, and not a few are convinced that this ought to be followed by the injection of an appropriate serum.

### THIS WEEK'S DRUG MARKET.

Business continues fairly active, although, of course, the extravagant prices ruling for so many medicinal products tends to restrict the demand for them. Among the articles that have advanced in price since last report was written are eserine, butylchloral hydrate, santonin, salicin, phenacetin, and the benzoates. The most notable advance is in the case of salicin, this drug being 33 per cent. dearer. An advance in the price of camphor is by no means improbable. The very high prices asked for cod-liver oil continue to keep buyers off the market; it is really difficult to know what to do. Although it is not impossible that prices will decline to some slight extent, it is just as likely that they will advance, and in any case there does not appear to be much likelihood of a substantial reduction. To those who have been accustomed to paying something like a quarter the present quotation in normal times it is no easy matter to regard the high figures asked as reasonable; but, on the other hand, the prospects of a reduction in the near future seem to be very faint indeed. Chamomiles continue very scarce and dear. The value of senega is not quite so firmly maintained, and the same applies to ergot of rye. Epsom salts are coming on the market more freely and lower prices will probably prevail before long. Opium is unchanged, and there are no alterations in the prices of morphine and codeine. For the small quantities of thymol available extremely high prices are asked.

### TO OUR READERS.

Contributions are specially invited from any of our readers to these columns. They should deal with topical subjects and news. They must be authenticated for the information of the Editor only. The minimum payment if published is 5s. There is no hard-and-fast rule as to space, but notes of about twenty lines in length are preferred.

